# COVID-19 and the rise of virtual medicine in spine surgery: a worldwide study

**DOI:** 10.1007/s00586-020-06714-y

**Published:** 2021-01-16

**Authors:** Peter R. Swiatek, Joseph A. Weiner, Daniel J. Johnson, Philip K. Louie, Michael H. McCarthy, Garrett K. Harada, Niccole Germscheid, Jason P. Y. Cheung, Marko H. Neva, Mohammad El-Sharkawi, Marcelo Valacco, Daniel M. Sciubba, Norman B. Chutkan, Howard S. An, Dino Samartzis

**Affiliations:** 1 Department of Orthopaedic Surgery, Northwestern University, Chicago, IL USA; 2 Department of Orthopaedic Surgery, Hospital for Special Surgery, New York, NY USA; 3 Department of Orthopaedic Surgery, Rush University Medical Center, Orthopaedic Building, Suite 204-G, 1611 W Harrison Street, Chicago, IL 60612 USA; 4 The International Spine Research and Innovation Initiative, Rush University Medical Center, Chicago, IL USA; 5 Research Department, AO Spine International, Davos, Switzerland; 6 Department of Orthopaedics and Traumatology, The University of Hong Kong, Hong Kong, China; 7 Department of Orthopaedic and Trauma Surgery, Tampere University Hospital, Tampere, Finland; 8 Department of Orthopaedic and Trauma Surgery, Assiut University Medical School, Assiut, Egypt; 9 Department of Orthopaedics, Churruca Hospital de Buenos Aires, Buenos Aires, Argentina; 10 Department of Neurosurgery, John Hopkins University, Baltimore, MD USA; 11 Department of Orthopaedic Surgery, University of Arizona College of Medicine, Phoenix, AZ USA; 12 Neuroscience Institute, Virginia Mason, Seattle, WA USA

**Keywords:** Telehealth, Telemedicine, Online education, Virtual medicine, Spine surgery, COVID-19, Coronavirus, New technologies

## Abstract

**Purpose:**

The COVID-19 pandemic forced many surgeons to adopt “virtual medicine” practices, defined as telehealth services for patient care and online platforms for continuing medical education. The purpose of this study was to assess spine surgeon reliance on virtual medicine during the pandemic and to discuss the future of virtual medicine in spine surgery.

**Methods:**

A comprehensive survey addressing demographic data and virtual medicine practices was distributed to spine surgeons worldwide between March 27, 2020, and April 4, 2020.

**Results:**

902 spine surgeons representing seven global regions responded. 35.6% of surgeons were identified as “high telehealth users,” conducting more than half of clinic visits virtually. Predictors of high telehealth utilization included working in an academic practice (OR = 1.68, *p* = 0.0015) and practicing in Europe/North America (OR 3.42, *p* < 0.0001). 80.1% of all surgeons were interested in online education. Dedicating more than 25% of one’s practice to teaching (OR = 1.89, *p* = 0.037) predicted increased interest in online education. 26.2% of respondents were identified as “virtual medicine surgeons,” defined as surgeons with both high telehealth usage and increased interest in online education. Living in Europe/North America and practicing in an academic practice increased odds of being a virtual medicine surgeon by 2.28 (*p* = 0.002) and 1.15 (*p* = 0.0082), respectively. 93.8% of surgeons reported interest in a centralized platform facilitating surgeon-to-surgeon communication.

**Conclusion:**

COVID-19 has changed spine surgery by triggering rapid adoption of virtual medicine practices. The demonstrated global interest in virtual medicine suggests that it may become part of the “new normal” for surgeons in the post-pandemic era.

## Introduction

Within weeks, the COVID-19 pandemic became one of the greatest global health crises of our time [1]. Excessive strain on the health care system prompted hospitals and practices around the world to pivot in an effort to stop the spread of the COVID-19 and to treat the acutely ill [2]. With the unprecedented redirection of staff and resources toward management of COVID-19 and seemingly universal order to *shelter in place* [3], subspecialty services such as orthopedic and neurological spine surgery have had to adapt their service offerings to ensure that their patients continued to receive the necessary care. One of the most significant changes has been the rapid adoption and acceleration of “virtual medicine” practices, defined as reliance on telehealth services [4] to facilitate surgeon-to-patient interfacing and use of online educational platforms [5] to support continued physician learning during these times of social distancing [6].

Telehealth involves the use of advanced audiovisual aids to provide care for patients or facilitate physician-to-physician collaboration [7]. Previously considered more of an adjunct service with low reimbursement rates prior to Spring 2020, telehealth capabilities were firmly established in only about half of the US hospitals [7]. Now, with the rise of COVID-19 and increased pressures to treat patients virtually, government agencies are expanding reimbursements to cover telehealth visits, and health care systems are rapidly developing more sophisticated telehealth capabilities [8]. In addition to implementing telehealth services, spine surgeons have swiftly adopted online education as a primary means of continued surgeon-to-surgeon engagement during a time widespread elective case stoppage, in-person gathering restrictions, and travel constraints [9]. Early online education offerings during the pandemic focused primarily on dissemination of information about COVID-19 and its impact on the orthopedic and neurological surgery communities. Online education opportunities rapidly expanded to include local topic-based spine webinars—including journal clubs, case discussions, and indication conferences—to larger spine conferences hosted by professional societies such as AO Spine, the North American Spine Society, and the Scoliosis Research Society, among others [5, 10–12].

Despite anecdotal reports that surgeons and health systems are increasingly reliant on technology for surgeon-to-patient engagement and for continuing surgical education—so-called virtual medicine practices—the degree to which virtual medicine has been integrated into the practice of spine surgeons is unknown. The aims of this worldwide survey-based study are (1) to assess the degree to which spine surgeons worldwide have integrated telehealth services into their practice, (2) to determine the interest in online learning and physician engagement platforms for continuing spine surgeon education, and (3) to discuss how this rapid expansion of these virtual medicine practices may impact spine surgery care in a post-pandemic world.

## Materials and methods

### Survey design and distribution

The AO Spine COVID-19 and Spine Surgeon Global Impact Survey were developed to assess the spine surgeons’ perspective of the impact of COVID-19 on spine surgery practice, education, and outlook [13]. Questions were prepared via the Delphi method, in which multiple board-certified attending spine surgeons reviewed the appropriateness of each question prior to achieving consensus [14]. Scope of the survey included surgeon demographics/characteristics, impact on practice, and utilization of online platforms for patient care, physician-to-physician communication, and education.

The survey was distributed in English via email to the AO Spine membership who had previously elected to receive surveys for academic purposes. AO Spine members were selected as the target audience, as this group represents the largest international society of spine surgeons in the world. On March 27, 2020, the survey was emailed to 3805 members of AO Spine, representing approximately more than 60% of the total AO Spine membership. Respondents were given a total of nine days to complete the survey prior to the pre-determined end date of April 4, 2020. Each participant was informed that their participation in the survey was completely voluntary; that they could end their participation at any time; and that their response would likely anonymously be aggregated and analyzed for dissemination in peer-reviewed journals, educational and instructional media, or social media.

### Statistical analyses

Analyses were performed using Stata version 13.1 (StataCorp LC, College Station, TX). Means and percentages were made for rank order and count data, respectively. The analysis focused on surgeons as individuals; surgeons as “high telehealth” users, defined as more than 50% of clinic visits conducted using telehealth platforms; surgeons as having increased interest in online education; and “virtual medicine” surgeons, defined as reporting both high telehealth utilization and increased interested in online education platforms. Fisher’s exact tests and Chi-squared tests were utilized to determine difference in count data where applicable. Three separate multivariate regressions were conducted to determine predictive factors of being a high telehealth user, expressing increased interest in online education, and being a virtual medicine surgeon. Independent variables used in the multivariate analyses were selected based upon significance determined in the aforementioned between group analyses. Results of the multivariate analyses were reported using odds ratios (OR) with OR = 1 indicating no difference between independent and dependent variables. 95% confidence intervals (CI) were also calculated to assess precision of the risk estimate. A *p* value of < 0.05 was used to determine statistical significance.

## Results

902 spine surgeons from 91 countries and 7 regions responded to this survey. European spine surgeons comprised the largest group by region (242/881, 27.5%). Surgeons from Asia (213/881, 24.2%) and North America (152/881, 17.3%) were the second and third most represented groups by regions, respectively (Table 1).

**Table 1:** Survey respondent demographics stratified by telehealth usage, interest in online education, and status as a virtual medicine surgeon

	All respondents (*n* = 902)	> 50% telehealth^a^ (*n* = 285)	≤ 50% telehealth (*n* = 516)	*p* value	More interested in online education (*n* = 601)	Less interested in online education (*n* = 126)	*p* value	Virtual medicine surgeon^b^ (*n* = 211)	Non-virtual medicine surgeon^b^ (*n* = 595)	*p* value
Age				0.508			0.03			0.297
25–34 years	130 (14.5)	42 (14.8)	70 (13.6)		82 (13.7)	14 (11.1)		33 (15.7)	79 (13.3)	
35–44 years	344 (38.4)	113 (36.8)	197 (38.2)		244 (40.7)	39 (31)		86 (41)	227 (38.2)	
45–54 years	245 (27.4)	82 (29.9)	137 (26.6)		156 (26)	48 (38.1)		57 (27.1)	164 (27.6)	
55–64 years	150 (16.8)	39 (13.7)	96 (18.6)		100 (16.7)	24 (19.1)		26 (12.4)	109 (18.3)	
65 +	26 (2.9)	8 (2.8)	16 (3.1)		18 (3)	1 (0.8)		8 (3.8)	16 (2.7)	
Sex				0.046			0.449			0.011
Female	55 (6.2)	23 (8.2)	24 (4.7)		40 (6.8)	6 (4.9)		20 (9.7)	28 (4.8)	
Male	826 (93.8)	256 (91.8)	484 (95.3)		551 (93.2)	116 (95.1)		186 (90.3)	558 (95.2)	
Estimated home city population				0.0006			0.315			0.0012
< 100,000	46 (5.2)	6 (2.1)	35 (6.8)		28 (4.7)	7 (5.6)		4 (1.9)	37 (6.2)	
100,000–500,000	185 (20.7)	73 (25.6)	96 (18.7)		136 (22.6)	25 (19.8)		57 (27)	113 (19.1)	
500,000–1,000,000	136 (15.2)	56 (19.7)	68 (13.2)		101 (16.8)	14 (11.1)		43 (20.4)	81 (13.7)	
1,000,000–2,000,000	144 (16.1)	38 (13.3)	89 (17.3)		98 (16.3)	19 (15.1)		31 (14.7)	96 (16.2)	
> 2,000,000	382 (42.8)	112 (39.3)	226 (44)		238 (39.6)	61 (48.4)		76 (36.0)	266 (44.9)	
Geographic region				< 0.0001			0.023			< 0.0001
Africa	44 (5)	9 (3.2)	32 (6.3)		31 (5.2)	7 (5.7)		6 (2.9)	35 (6)	
Asia	213 (24.2)	38 (13.5)	147 (28.9)		132 (22.3)	35 (28.2)		29 (14)	157 (26.7)	
Australia	8 (0.9)	2 (0.7)	6 (1.2)		7 (1.2)	1 (0.8)		2 (1)	6 (1)	
Europe	242 (27.5)	91 (32.4)	132 (25.9)		160 (27)	41 (33.1)		68 (32.9)	155 (26.4)	
Middle east	77 (8.7)	13 (4.6)	53 (10.4)		54 (18.2)	6 (4.8)		9 (4.4)	59 (10)	
North America	152 (17.3)	94 (33.5)	49 (9.6)		108 (18.2)	27 (21.8)		66 (31.9)	79 (13.4)	
South/Latin America	145 (16.5)	34 (12.1)	90 (17.7)		100 (16.9)	7 (5.7)		27 (13)	97 (16.5)	

^a^Definition of “high telehealth” user

^b^Virtual medicine surgeons are defined as > 50% telehealth usage and expressed interest in online spine education

Approximately half of all surveyed surgeons (402/800, 50.3%) were using telehealth for at least 25% of clinic visits and 35.6% (285/801) were “high telehealth surgeons,” conducting more than half of visits virtually. The majority of high telehealth surgeons are between 35 and 54 years of age (195/284, 68.7%) and practice in Europe (91/281, 32.4%) or North America (94/281, 33.5%). Surgeons interested in online education are primarily between 35 and 54 years of age (400/600, 66.7%) and practice in Europe (160/593, 27.0%) or Asia (132/595, 22.3%). Virtual medicine surgeons, defined as those with > 50% telehealth usage and interest in online spine education, are between 35 and 54 years of age (143/210, 68.1%), practice in large cities with more than one million people (107/211, 50.7%), and live in Europe (68/ 207, 32.9%) and North America (66/207, 31.9%).

**Fig. 1:**
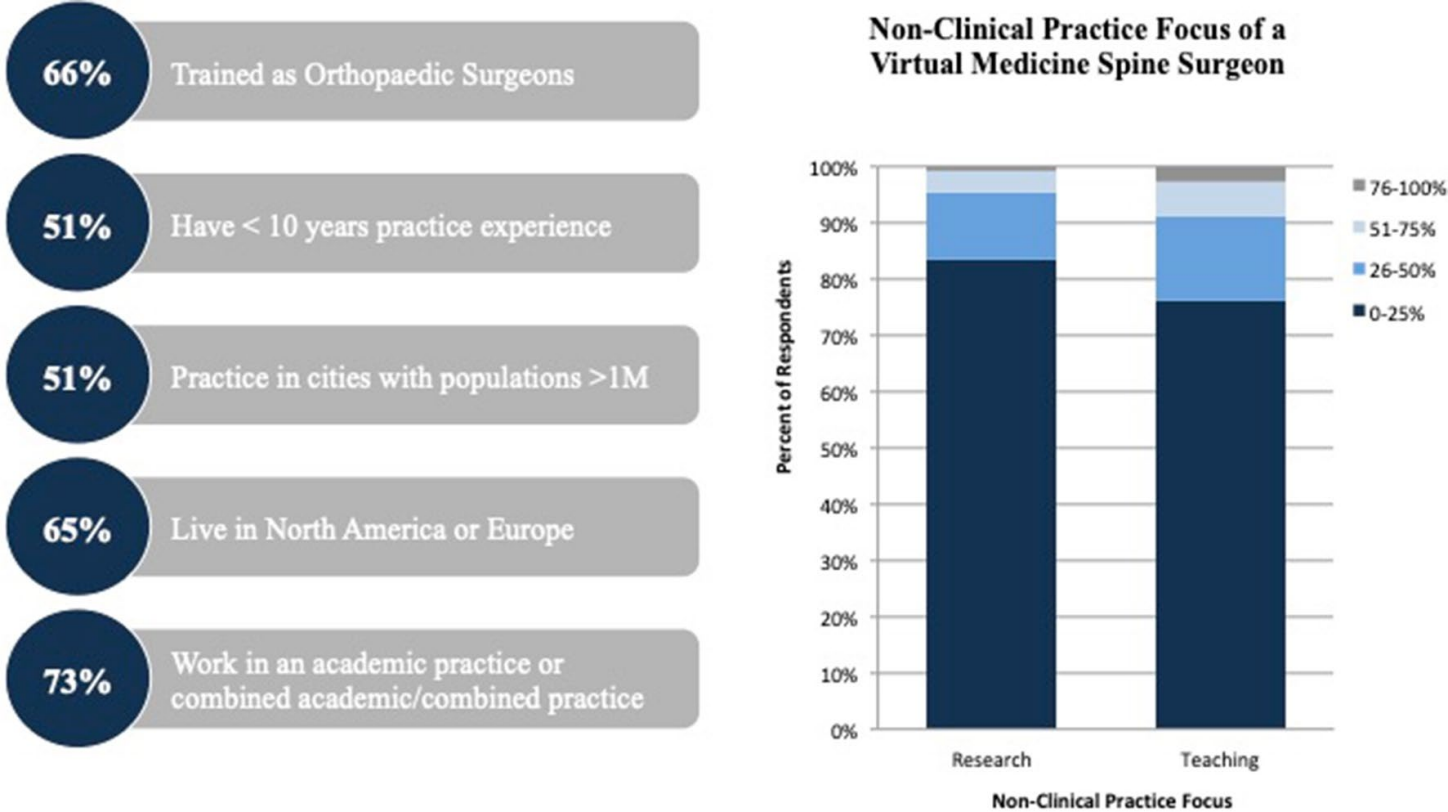
Profile of a virtual medicine spine surgeon

Table 2 highlights the practice demographics of spine surgeons who have integrated telehealth modalities into their practice. Most respondents were spine surgeons with orthopedic surgery backgrounds (637/902, 70.6%) who work primarily in academic or private/academic combined institutions. Nearly two-thirds of surgeons reported that their practice is more than 75% clinical (590/893, 66.1%) and the majority dedicated less than a quarter of their work to research (731/893, 81.9%). Academic surgeons, who comprised the largest portion of the respondents (405/802, 45.4%), relied heavily on telehealth, with 54.1% of their group conducting > 25% of visits virtually (*p* < 0.0001). There was no significant difference in usage of clinical telehealth based upon surgeon specialty, completion of fellowship, years in training, or type of practice (i.e., research, clinic, or teaching). Virtual medicine surgeons (Fig. 1) were primarily orthopedic surgeons (140/210, 66.4%), those with less than 10 years of practice experience (74/146, 50.7%), and those in academic practice (112/211 53.1%).

**Table 2:** Survey respondent practice demographics stratified by telehealth usage, interest in online education, and status as a virtual medicine surgeon

	All respondents (*n* = 902)	> 50% telehealth^a^ (*n* = 285)	≤ 50% telehealth (*n* = 516)	*p* value	More interested in online education (*n* = 601)	Less interested in online education (*n* = 126)	*p* value	Virtual medicine surgeon^b^ (*n* = 211)	Non-virtual medicine surgeon^b^ (*n* = 595)	*p* value
Specialty				0.097			0.491			0.095
Neurosurgery	234 (25.9)	86 (30.2)	130 (25.2)		167 (27.8)	30 (23.8)		63 (29.9)	153 (25.7)	
Orthopedics	637 (70.6)	191 (67)	374 (72.5)		418 (69.6)	92 (73)		140 (66.4)	428 (71.9)	
Pediatric surgery	2 (0.2)	1 (0.4)	0 (0)		1 (0.2)	1 (0.8)		1 (0.5)	1 (0.2)	
Trauma	12 (1.3)	6 (2.1)	5 (1)		10 (1.7)	1 (0.8)		6 (2.9)	5 (0.9)	
Other	17 (1.9)	1 (0.4)	7 (1.4)		5 (0.8)	2 (1.6)		1 (0.5	8 (1.3)	
Fellowship trained				0.435			0.189			0.408
Yes	581 (72.5)	202 (70.9)	379 (73.5)		437 (72.7)	94 (74.6)		148 (70.1)	435 (73.1)	
No	220 (27.5)	83 (29.1)	137 (26.6)		164 (27.2)	32 (25.4)		63 (29.9)	160 (26.9)	
Year since training completion				0.648			0.036			0.117
< 5 years	161 (25.3)	59 (59.4)	90 (23.9)		119 (27.5)	13 (13.7)		47 (32.2)	102 (23.5)	
5–10 years	141 (22.2)	40 (19.9)	85 (22.6)		92 (21.3)	25 (26.3)		27 (18.5)	100 (23)	
10–15 years	104 (16.4)	33 (16.4)	61 (16.2)		64 (14.8)	19 (20)		23 (15.8)	71 (16.4)	
15–20 years	117 (18.4)	34 (16.9)	74 (19.6)		77 (17.8)	23 (24.2)		20 (13.7)	88 (20.3)	
> 20 years	113 (17.8)	25 (17.4)	67 (17.8)		81 (18.7)	15 (15.8)		29 (19.9)	73 (16.8)	
Practice type				0.0001			0.013			0.088
Academic/private combined	204 (22.9)	58 (20.4)	132 (25.7)		145 (24.2)	27 (21.6)		41 (19.4)	149 (25.2)	
Academic	405 (45.4)	160 (56.3)	208 (40.5)		259 (43.2)	69 (55.2)		112 (53.1)	258 (43.6)	
Private	144 (16.1)	39 (13.7)	86 (16.7)		106 (17.7)	9 (7.2)		33 (15.6)	94 (15.9)	
Public/local hospital	139 (15.6)	27 (9.5)	88 (17.1)		90 (15)	20 (16)		25 (11.9)	91 (15.4)	
Practice breakdown										
*Percent research*				0.043			0.845			0.279
0–25%	731 (81.9)	235 (82.5)	425 (82.5)		494 (82.3)	103 (81.8)		176 (83.4)	487 (82)	
26–50%	129 (14.4)	35 (12.3)	80 (15.5)		90 (15)	18 (14.3)		25 (11.9)	91 (15.3)	
51–75%	21 (2.4)	12 (4.2)	7 (1.4)		11 (1.8)	3 (2.4)		8 (3.8)	11 (1.9)	
76–100%	12 (1.3)	3 (1.1)	3 (0.6)		5 (0.8)	2 (1.6)		2 (1)	5 (0.8)	
*Percent clinical*				0.329			0.053			0.094
0–25%	22 (2.5)	4 (1.4)	9 (1.7)		7 (1.2)	4 (3.2)		2 (1)	11 (1.9)	
26–50%	87 (9.7)	28 (9.8)	42 (8.1)		56 (9.3)	10 (7.9)		21 (10)	50 (8.4)	
51–75%	194 (21.7)	54 (19)	125 (24.2)		121 (20.1)	36 (28.6)		35 (16.6)	144 (24.2)	
76–100%	590 (66.1)	199 (69.8)	340 (65.9)		417 (69.4)	76 (60.3)		153 (72.5)	390 (65.6)	
*Percent Teaching*				0.891			0.168			0.788
0–25%	668 (74.9)	219 (77.1)	387 (75.2)		443 (74)	104 (82.5)		160 (76.2)	449 (75.6)	
26–50%	152 (17)	43 (15.1)	89 (17.3)		103 (17.2)	16 (12.7)		31 (14.8)	101 (17)	
51–75%	50 (5.6)	15 (5.3)	26 (5.1)		37 (6.2)	3 (2.4)		13 (6.2)	29 (4.9)	
76–100%	22 (2.5)	7 (2.5)	13 (2.5)		16 (2.7)	3 (2.4)		6 (2.9)	15 (2.5)	

^a^Definition of “high telehealth” user

^b^Virtual medicine surgeons are defined as > 50% telehealth usage and expressed interest in online spine education

Predictors of high telehealth usage, interest in online education, and status as a virtual medicine surgeon are displayed in Tables 2, 3, 4 and 5. Working in a purely academic practice (OR 1.68; 95% CI 1.22 to 2.31; *p* = 0.0015) or practicing in Europe/North America (OR 3.42; 95% CI 2.42 to 4.84; *p* < 0.0001) was associated with increased odds of being a high telehealth user (Table 3). With respect to online education and engagement platforms, having a practice that dedicated more than 25% to teaching (OR 1.89; 95% CI 1.04 to 3.43, *p* = 0.037) was the only factor significantly associated with increased odds of expressing interest in spine education (Table 4). Similar to predictors of high telehealth usage, living in Europe or North America (OR 2.28; 95% CI 1.49 to 3.51; *p* = 0.0002) and practicing in a purely academic practice (OR 1.72; 95% CI 1.15 to 2.58; *p* = 0.0082) were the only factors associated with increased likelihood of being a virtual medicine spine surgeon (Table 5).

**Table 3:** Multivariate analysis of > 50% telehealth usage

	Odds ratio (95% CI)	*p* value
Male sex	0.57 (0.31, 1.09)	0.088
Europe or North America region	3.42 (2.42, 4.84)	< 0.0001
City population > 1,000,000	1.06 (0.76, 1.48)	0.729
Orthopedic subspecialty	0.76 (0.54, 1.08)	0.125
Purely academic practice	1.68 (1.22, 2.31)	0.0015
Practice breakdown > 25% rsearch	1.34 (0.87, 2.06)	0.185

**Table 4:** Multivariate analysis of interest in online spine education

	Odds ratio (95% CI)	*p* value
Age < 45 years	1.23 (0.6, 2.53)	0.567
Europe or North America region	0.78 (0.48, 1.27)	0.32
Fellowship trained	4.4 (0.26, 72.9)	0.302
0–10 years since training completion	1.28 (0.6, 2.6)	0.5
Purely academic practice	0.64 (0.4, 1.02)	0.059
Practice breakdown < 75% clinical	0.67 (0.41, 1.1)	0.114
Practice breakdown > 25% teaching	1.89 (1.04, 3.43)	0.037

**Table 5:** Multivariate analysis of region, city, population, and practice type on status as a virtual medicine surgeon

	Odds ratio (95% CI)	*p* value
Male sex	0.53 (0.23, 1.24)	0.142
Europe or North America region	2.28 (1.49, 3.51)	0.0002
City population > 1,000,000	0.94 (0.62, 1.43)	0.769
0–10 years since training completion	0.94 (0.64, 1.42)	0.816
Purely academic practice	1.72 (1.15, 2.58)	0.0082
Practice breakdown < 75% Clinical	0.8 (0.51, 1.25)	0.326

^a^Virtual medicine surgeons are defined as > 50% telehealth usage and expressed interest in online spine education

**Fig. 2:**
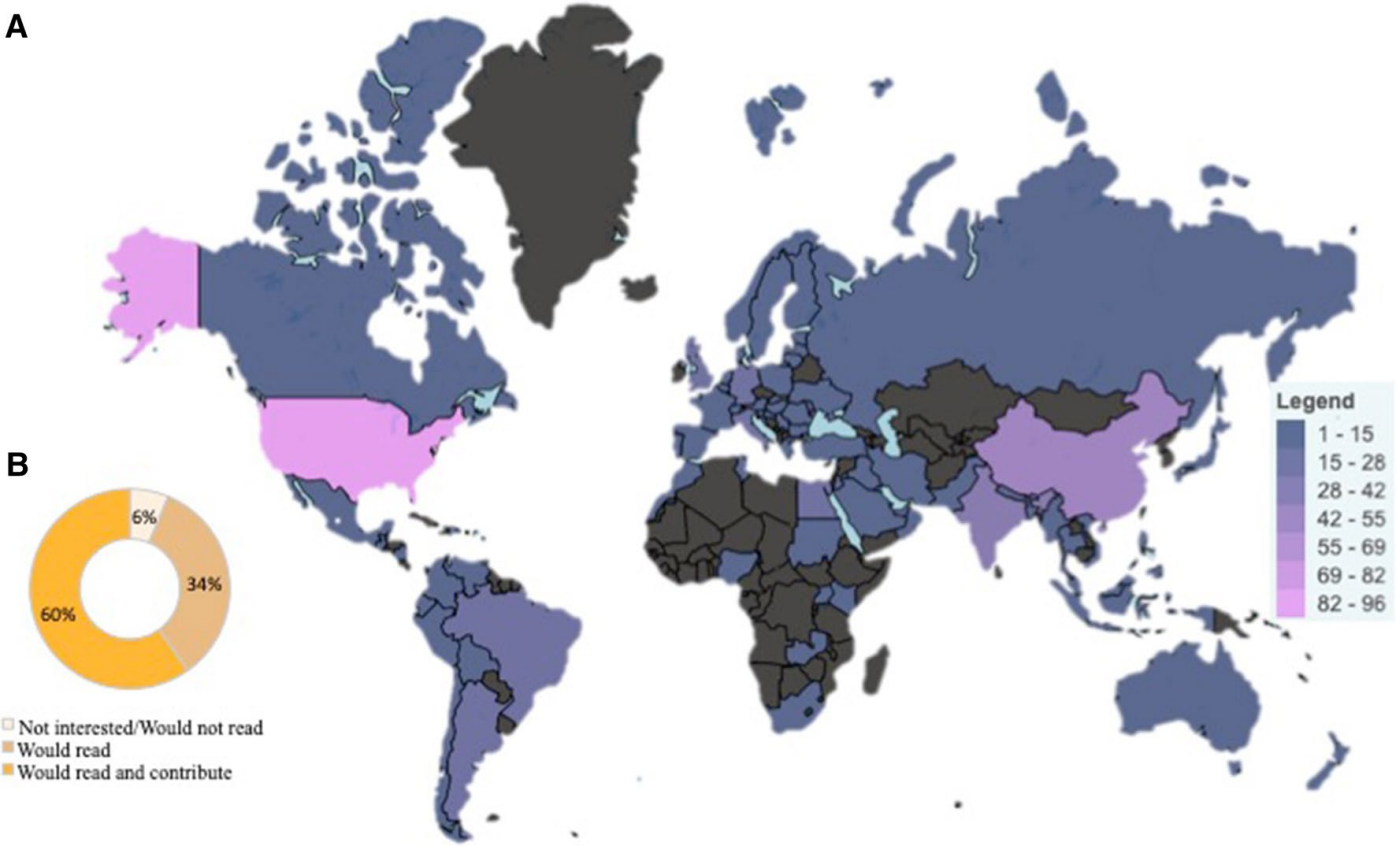
Spine surgeon interest in a centralized online engagement platform. **a** Geographic heat map of spine surgeons who expressed interest in an online blog or centralized online surgeon-to-surgeon engagement platform. **b** Breakdown of spine surgeons’ levels of interest in an online blog or centralized online surgeon-to-surgeon engagement platform

Most spine surgeons (686/732, 93.8%) expressed interested in a spine-specific platform through which surgeons could connect, collaborate, and seek support (Fig. 2). European and Asian spine surgeons represented the largest group at 25.5% (187/732) and 22.4% (164/732) of all surgeons, respectively. More than 93% of all surgeons outside of North America would be interested in a blog; however, 16.6% of North American surgeons reported that they would likely not participate in or read a blog. Academic surgeons (308/732, 42.1%) comprised the largest group of surgeons interested in a centralized platform to communicate. When compared to surgeons within the same practice type, more than 92.5% of surgeons reported interest in a blog.

## Discussion

To our knowledge, this is the largest study of its kind aimed at assessing the impact of COVID-19 on worldwide practice of virtual medicine, including telehealth use for patient care and online platforms for physician education. With more than 900 total respondents, we noted geographic, demographic, and practice-type variations in the general use of telehealth applications for spine surgery clinic visits. We found a high-rate of telehealth utilization among spine surgeons, with more than half of surgeons worldwide leveraging telehealth to conduct a significant number (> 25%) of virtual physician–patient interactions. Moreover, we found that spine surgeons worldwide is interested in continuing online education and connecting with other surgeons during global crises like the COVID-19 pandemic. More than 80% of surgeons reported interest in online education and more than 93% would utilize a centralized communication platform to communicate with other surgeons. Lastly, we identified 211 virtual medicine spine surgeons, who we can expect to lead the charge in the rise of telehealth and online education in the post-pandemic era.

Our findings support the newly published literature and press releases that have described the rapid acceleration of telehealth practices in orthopedic and neurosurgical practices in the wake of the COVID-19 pandemic. In a recent survey of 168 orthopedic surgery departments across the USA, Parisien et al. [15] found that 63% of institutions are providing telehealth services with 23% currently in the process of establishing new telehealth capabilities. Of those institutions with established telehealth services, more than 80% implemented these systems recently in response to the COVID-19 pandemic [15]. Similar to our results, these authors found that increased utilization of telehealth services was associated with academic institutions [15]. Several groups have already begun to evaluate the utility and safety of virtual clinic visits [16]. Two groups of orthopedic shoulder and elbow surgeons groups have published on their findings that most patients undergoing rotator cuff repair [17, 18] or shoulder arthroplasty [18] could have a safe and effective visit virtually, without direct surgeon-to-physician contact. In the realm of spine surgery, conducting a proper neurological exam has been a primary impediment to rapid adoption by spine surgeons. Several studies offer guidance on performance of a virtual physical examination, including specific exam maneuvers that can help spine surgeons assess physical function, motor strength, and sensation [19–23]. Although the maneuvers discussed in these studies can provide a fair impression of a patient’s musculoskeletal and neurological status, the need for a complete, validated, and pressure-tested virtual physical examination is apparent [24].

The US government has played a substantial role in facilitating the rapid roll-out of telehealth programs across the country. In mid-March of 2020, the Department of Health and Human Services (HHS), the Centers for Medicare & and Medicaid Services (CMS), and the HHS Office of Civil Rights (OCR) announced measures that would encourage physicians and surgeons to conduct telehealth visits, including temporarily expanding reimbursements for telehealth visits and relaxing restrictions on acceptable technology platforms [25]. This included the CMS 1135 Waiver [26], which broadened access to Medicare telehealth services and removed state licensing restrictions through the duration of the COVID-19 crisis. Several other governmental organizations have developed guidelines and programs to assist providers with implementation and funding of telehealth services [27], including the National Consortium of Telehealth Resource Centers (NCTRC) [27].

To further the cause, the Federal Communications Commission (FFC) approved $200 million in early April to equip hospitals and health care providers with telehealth technology and implementation support [28]. Although all sources point toward continued governmental support for telehealth services during the pandemic, no one can predict exactly how support may change as practices return to *business as usual*. Potential interventions include medical boards scaling back on interstate licensure flexibility [29] and CMS narrowing the scope of reimbursable activities [5]. Cost will continue to play a major role in the future development of telehealth capabilities. Traditional capital expenditure models, including hardware, software, security platforms, and systems implementation, can start around $42,000 and scale up depending size of practice and specific clinical needs [30]. Importantly, these initial start-up figures often do not include the full costs of maintenance and continuing improvement such as software upgrades, equipment upgrades, and information technology support. To curb the weight of initial start-up costs, some companies have begun offering flat fee services starting anywhere from $49 for a simple single-provider HIPAA compliant web application[31] to more than $800 per month flat fee, which covers workstation, exam camera, and access to the integrated telehealth software without additional per provider costs [30]. As demand for long-term telehealth platforms continue to expand in the post-pandemic era, we expect to see competitive service offerings aimed at lowering financial barriers, allowing a more ubiquitous adoption of health technology.

Although the seemingly ubiquitous demand for telehealth services has spiked across the globe, the feasibility and capability of implanting such services across regions are much more variable. A recent publication by the COVID-19 Pandemic Health System Resilience Program (REPROGRAM) international consortium reviewed this variability in national telehealth programs across various countries/regions with the intent of identifying differences in current telehealth frameworks, gaps in implementation, and future directions for improvement [32]. Prior to the pandemic, countries in Western Europe, such as the UK [33] and France [34], had established systems for moving in-person medical consultations to virtual consultations. The pandemic challenged these health systems by testing the scale of their telehealth infrastructure, by necessitating governmental funding for growth of current telehealth offerings, and by highlighting the need for better communication among national health systems [32].

Countries with less-developed health systems face a different set of challenges with respect to telehealth. For example, many African nations, particularly those in Sub-Saharan Africa, face challenges such as connectivity issues, device ownership, physician shortages, ongoing political conflicts, and lack of governmental support for technological innovation [32]. Overcoming these barriers will likely require intervention at the patient, physician, system, and government level in addition to international support.

At the patient-level, even well-established health care systems will face challenges related to patient computer literacy, patient access to high-quality telehealth compatible devices, and patient access to reliable internet coverage [35]. For example, in the USA, it is estimated that 31.8 million Americans do not have sufficient comfort or competence with technology to use a computer [36]. Moreover, barriers to access disproportionately affect older and underrepresented populations. For example, more than half of US households headed by individuals older than 65 years of age do not have a smartphone and one-third do not have a desktop or laptop [37]. Widespread adoption of telehealth services may be limited to certain populations unless barriers such as digital literacy and access to telehealth-capable technology are addressed.

With respect to online education and the impact on physician-to-physician communication and education, we found that the majority of spine surgeons (> 80%) are interested in participating in online spine education and/or and AO spine-specific blogging. This desire for more virtual learning opportunities echoes the general attitude that regular surgeon engagement, even virtual, facilitates continuous surgeon education and ultimately increases surgeon competency. In their review of the virtual learning environment, Palan et al. [38] discusses how the virtual learning environment can actually enhance surgeon learning. Specifically, learners can easily reference digitally recorded sessions for clarification on difficult to understand topics [38]. Kogan et al. [39] make specific recommendations regarding the use of various team-based platforms, such as Microsoft Teams and Zoom, in an effort to increase team-based learning efforts. In accordance with the results of our survey, participation in these virtual conferences is generally higher than normal in-person conferences [39].

Moving forward, online learning will likely become paramount in the continuing education of spine surgeons around the world. In the short-term, medical groups and societies will likely transform more face-to-face events into virtual lectures and online conferences. In the post-pandemic era, large national and regional spine meetings, once a staple of educational camaraderie, may be transformed into virtual online webinars. Industry-sponsored meetings and exhibitions may digitally convert to three-dimensional virtual learning experiences for spine surgeon participants. In the more distant future, simulators and virtual reality (VR) platforms, although in their infancy, may become more sophisticated and eventually provide the visual and tactile feedback necessary to mimic surgical reality. Online education provides the added benefit of “learning on demand,” decreased travel and accommodation costs, more efficient utilization of time, and the opportunity to learn at the comfort of one’s home or office while connecting with international colleagues. Several mediums have already started to promote online spine education, such as the eccElearning^29^ and other academic/society outlets, offering postgraduate certificates, diplomas, and other certifications.

This survey-based study is not without limitation. First, the questionnaire was only disseminated among spine surgeons registered as AO Spine members who previously elected to participate in survey research (*n* = 3,805), and the overall response—although high in absolute number (*n* = 902)—was relatively low in rate (23.7%). In absolute terms, this represents approximately 15% of all AO Spine members. Low response rate does not necessarily indicate low validity; however, it may allow for certain biases to affect the generalizability of the findings. Spine surgeons who are active email users may be have been more likely to respond to the survey and AO Spine respondents may be more representative of an academic population. For example, of all 902 respondents, the majority were spine surgeons working in academic practices (45.4%, *n* = 405) or spines surgeons working in combined academic/private practices (22.9%,* n* = 204) (Table 1). Additionally, although the study leveraged the Delphi method for construction of the survey, time constraints imposed by the pandemic and the urgency with which the survey was launched prevented our group from conducting the usual pilot study for external validation. Additionally, survey questions aimed at assessing reliance on telehealth, online education, and online surgeon-to-surgeon engagement were high-level. Additional topic-specific questions would undoubtedly add granularity to the analysis and deepen our understanding of the pros and cons of virtual medicine practices. Despite these limitations, the input from responding spine surgeons from around the world sheds a new light on the current role of technology in patient care and physician education and provides a foundational understanding about the future of the virtual medicine spine surgeons.

## Conclusions

Without a doubt, COVID-19 has triggered a complete restructuring of how spine surgeons conduct their clinical practice. Telehealth, which was once considered technologically cumbersome, financially unproductive, and difficult from a medico-legal perspective, has now become part of clinical spine surgery. Moreover, medical education platforms, which were previously used intermittently, have now gained more interest by the community and become primary sources for online spine education and physician-to-physician communication. As we move through this COVID-19 crisis and into the post-pandemic era, we are hopeful for a return to some level of *normalcy*. However, we can expect that our newfound technological capabilities and faith in telehealth may elevate the practice of spine surgery to a *new normal*, to benefit both patients and surgeons alike.
